# LncRNA SNHG15 acts as a ceRNA to promote breast cancer progression through the miR-153-3p/KLF5 signal axis

**DOI:** 10.7150/jca.116946

**Published:** 2025-07-28

**Authors:** Xu Zheng, Luyao Zhang, Ying Feng, Fan Zhu, Jiangming Shi, Yang Feng, Qian Chen, Ruizhi Shen

**Affiliations:** 1 Department of Intensive Care Unit, Suzhou Hospital, Affiliated Hospital of Medical School, Nanjing University, Suzhou, China.; 2 Department of Oncology, The Affiliated Suzhou Hospital of Nanjing Medical University, Suzhou Municipal Hospital, Gusu School, Nanjing Medical University, Suzhou, Jiangsu, China.; 3 Department of Oncology, Wuxi No. 2 People's Hospital, Jiangnan University Medical Center, Wuxi 214002, China.; 4 Department of Central Laboratory, The Affiliated Suzhou Hospital of Nanjing Medical University, Suzhou Municipal Hospital, Gusu School, Nanjing Medical University, Suzhou, Jiangsu, China.

**Keywords:** SNHG15, miR-153-3p, KLF5, competing endogenous RNA, breast cancer

## Abstract

**Background:** Increasingly evidence shows that the interaction between long non-coding RNAs (lncRNAs), microRNAs (miRNAs) and their downstream target genes plays a pivotal role in the onset and progression of tumors, emerging as a focal point in tumor research. This study sought to assess the biological function of lncRNA SNHG15 and investigate the underlying mechanisms involved in SNHG15/miR-153-3p/KLF5 signal axis in breast cancer (BC).

**Methods:** The expressions of SNHG15, miR-153-3p and KLF5 in human BC tissues and cell lines were detected by quantitative real-time PCR and/or western blot. To investigate the biological functions of SNHG15, we knocked it down in BC cells and observed its effects both *in vitro* and *in vivo*. The underlying mechanisms of competitive endogenous RNA (ceRNA) between SNHG15 and miR-153-3p were elucidated through bioinformatics analysis, dual-luciferase reporter assays and rescue experiments.

**Results:** SNHG15 expression was notably elevated in BC tissues and cell lines. Knockdown of SNHG15 significantly reduced the ability of proliferation, migration and invasion in BC cells. miR-153-3p was a direct target of SNHG15, while miR-153-3p mediated the expression of KLF5 in BC cell lines. In addition, the effect of SNHG15 downregulation on the biological behavior of BC cells can be offset by the inhibition of miR-153-3p. Mechanically, SNHG15 may act as the ceRNA of miR-153-3p, thereby regulating the expression of its target gene KLF5.

**Conclusions:** SNHG15 promotes proliferation and metastasis by sponging miR-153-3p and regulates KLF5 expression, suggesting that SNHG15 may be a potential biomarker and therapeutic target for BC.

## Introduction

Breast cancer (BC) remains the primary malignant tumor threatening women's health in the world, with its incidence is increasing year by year 1-3. According to the latest World Health Organization (WHO) data, BC has surpassed lung cancer as the most prevalent cancer diagnosed among women worldwide. In recent years, despite advancements in diagnostic methods and early screening initiatives that have lowered BC mortality rates, many patients still face advanced-stage diagnosis or recurrence with metastasis after treatment, leading to a poor prognosis 4. Meanwhile, the pathogenesis of BC has not yet been fully understood. The research of BC key genes is of great significance for finding biomarkers of early BC, formulating targeted treatment strategies and improving patient survival rates 5, 6.

In recent years, with the development of high-throughput sequencing technology, increasing long non-coding RNAs (lncRNAs) have been discovered 7. Studies have shown that lncRNA plays an important role in cell physiological and pathological activities and participates in the occurrence and development of many diseases including tumors 8-10. While increasing lncRNAs have been confirmed to participate in the occurrence and development of BC as a tumor suppressor or oncogene, there is a scarcity of reports regarding the role of lncRNA SNHG15 (small nucleolar RNA host gene 15) in this context 10-15.

This study aims to elucidate the function and mechanism of lncRNA SNHG15 in the occurrence and development of BC. Our findings may introduce a novel molecular marker for clinical BC diagnosis and provide a foundation for targeted BC therapies and the development of new anticancer drugs.

## Materials and Methods

### Clinical tissue specimens

A total of 42 patients who underwent surgery and were confirmed as BC according to pathological results were included in the study. Portions of their tumor and adjacent nontumor tissues were immediately frozen in liquid nitrogen. The protocol for this study was approved by the Affiliated Suzhou Hospital of Nanjing Medical University. All patients signed the informed consent form. The clinical and pathological details are summarized in **Table 1**.

### Cell lines and culture

Human normal human breast epithelial cells (MCF-10A) and breast cancer cell lines (MCF-7, MDA-MB-231) were acquired from the Type Culture Collection of the Chinese Academy of Sciences (Shanghai, China). MCF-10A was grown in Mammary Epithelial Cell Growth Medium (MEGM BulletKit) (Lonza, Swiss). MCF-7 and MDA-MB-231 were grown in L-15 medium (Gibco, USA) supplemented with 10% fetal bovine serum (Gibco, USA). All cells were maintained in a humidified 37℃ incubator with an atmosphere containing 5% CO_2_.

### RNA isolation and quantitative real-time PCR analysis (qRT-PCR)

Total RNA was extracted from tissues or cells using the Trizol reagent kit (Invitrogen, USA). cDNA was synthesized using a reverse transcription kit (Takara, Japan). Real-time PCR analyses were performed with a Power SYBR Green PCR kit (Toyobo, Japan). SNHG15, miR-153-3p, and KLF5 (Kruppel-like factor 5) expression levels were normalized to GADPH or U6, respectively. All qRT-PCR reactions and data acquisitions were conducted on the Light Cycler480II Real-Time PCR Detection system (Roche, Switzerland).

### Virus infection and cell transfection

Recombinant lentiviruses expressing the sh-SNHG15 were obtained from GENEWIZ (Suzhou, China). BC cells stably transfected with these viruses were selected using 1.5 μg/ml puromycin (Calbiochem, USA) for 2 weeks. The miR-153-3p mimic, miR-153-3p inhibitor, and negative control (NC) oligonucleotides were purchased from GENEWIZ (Suzhou, China). Oligonucleotides and plasmids were transfected by Lipofectamine 3000 (Invitrogen, USA) according to the manufacturer's instructions.

### Cell proliferation assay

A cell proliferation assay was performed with a CCK-8 kit (Beyotime Biotechnology, China) according to the manufacturer's instructions. For the colony formation assay, a certain number of cells were placed into each well of a six-well plate and maintained in complete media for 2 weeks. The medium was replaced every 4 days. Colonies were fixed with methanol and stained with 0.1% crystal violet (Sigma-Aldrich, USA). The colony formation was determined by counting the number of stained colonies.

### Cell apoptosis

For the apoptosis assay, treated cells were stained with fluorescein isothiocyanate (FITC)-Annexin V and propidium iodide (PI) using an Annexin V-FITC/PI Apoptosis Detection Kit (Beyotime Biotechnology, China). After double staining with FITC and PI, the cells were analyzed by fluorescence-activated cell sorting (FACS) analysis. FACS was performed using CytoFLEX (Beckman Coulter, USA).

### Cell migration and invasion assays

Transwell assay was performed to assess the migration and invasion potential of BC Cells in vitro. For the migration assay, using 24-well culture plates with 8 μm pore-containing membrane inserts (Corning, China), the serum-free cell-containing medium was added to the upper chamber and the lower chamber contained medium supplemented with 15% FBS. Unlike the migration experiment, the upper chamber of the invasion assay was pretreated with Matrigel. Finally, cells below the membrane were stained with 0.4% trypan blue (Beyotime Biotechnology, China) and counted under a light microscope.

### Xenografted tumor model

Female athymic BALB/c nude mice (4-6 weeks old) were bought from Zhao Yan New Drug Research Center (Suzhou, Jiangsu, China) and maintained in laminar flow cabinets under SPF conditions. All animal-related experiments adhered to institutional guidelines and were approved by the Ethics Committee of the Affiliated Suzhou Hospital of Nanjing Medical University. 2*10^6^ MDA-MB-231 cells or MCF-7 cells that were stably transfected with sh-NC or sh-SNHG15 were subcutaneously injected into the flank of mice to establish BC xenografted tumor model.

### Dual-luciferase reporter assay

Full-length SNHG15 and KLF5 3'UTR sequences with wild-type or mutant miR-153-3p binding sites were inserted into pGL3 vectors. HEK293T cells were co-transfected with luciferase reporter plasmids and negative control (miR-NC) or miR-124-3p mimics using Lipofectamine 3000. A double-luciferase report system (Promega, USA) was used to determine the luciferase activity at 48 h post-transfection.

### Statistical analysis

The statistical analysis was performed using SPSS 19.0 software (IBM Corporation, USA). Student's t-test was used for data analysis to evaluate the statistical significance between treatment groups. A *P*-value of <0.05 was considered statistically significant.

## Results

### SNHG15 was upregulated in BC tissues and cell lines

**Figure 1:**
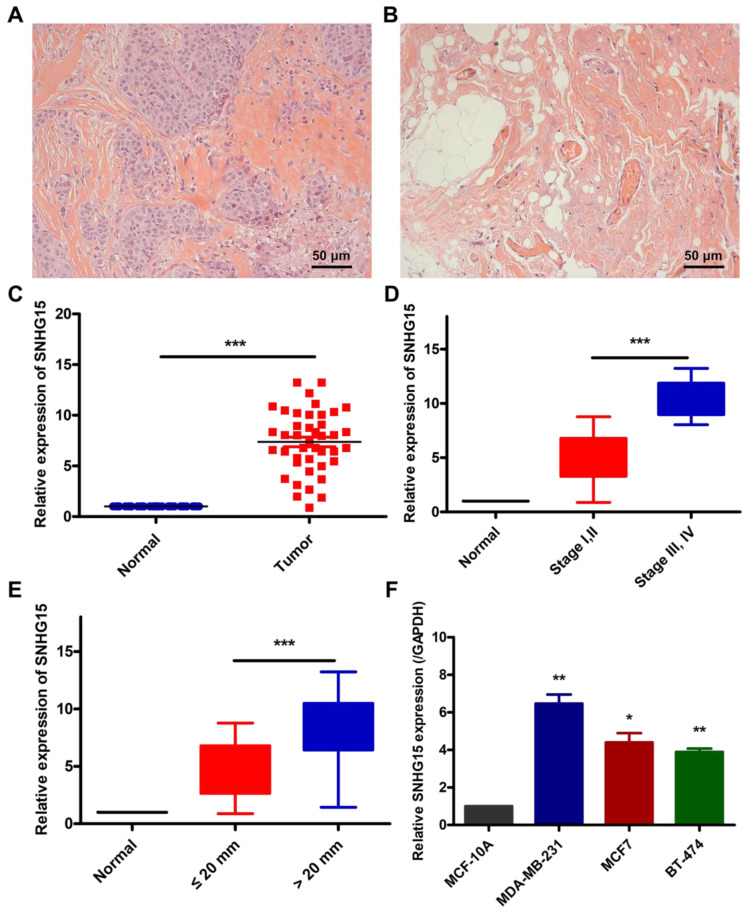
**Relative SNHG15 expression in BC tissues and its clinical significance.** A, Representative H&E staining of BC tissues. B, Representative H&E staining of the tissues adjacent to BC. C, Relative expression of SNHG15 in BC tissues in comparison with corresponding non-tumor normal tissues. D, SNHG15 upregulation correlated with larger tumor size. E, SNHG15 upregulation correlated with advanced pathological stage. F, SNHG15 expression levels of BC compared with normal human breast epithelial cells. *P < 0.05, **P < 0.01, ***P < 0.001.

We conducted qRT-PCR to assess SNHG15 expression in 42 pairs of BC tissues compared with adjacent normal tissues. **Figure 1A and B** are representative H&E staining of breast cancer tissues and normal breast tissue. As shown in **Figure 1C**, the SNHG15 expression level in BC tissues was significantly higher than that in the corresponding normal tissues. Similarly, compared with normal human breast epithelial cell MCF-10A, the expression of SNHG15 in MCF-7 or MDA-MB-231 is also higher **(Figure 1F)**. What's more, upregulated SNHG15 was correlated with larger tumor size and advanced TNM stage **(Figure 1D, E)**. These data indicate that SNHG15 may play the role of an oncogene in the process of BC progression.

### SNHG15 mediated cell proliferation, apoptosis, migration and invasion of BC

**Figure 2:**
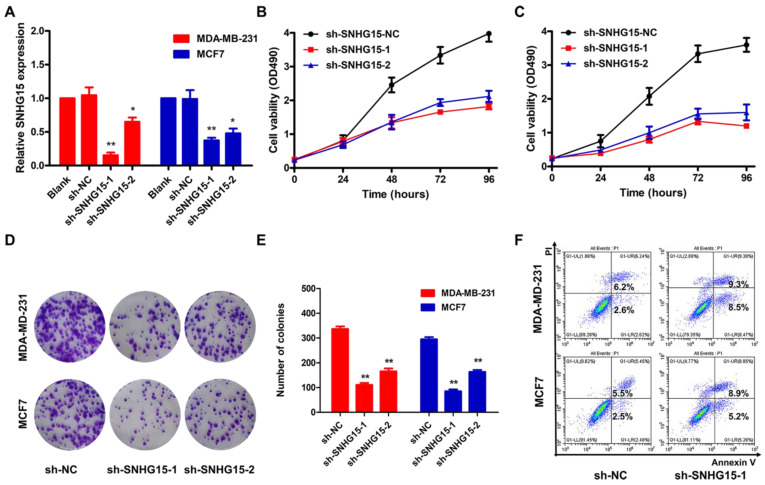
** SNHG15 mediated cell proliferation, apoptosis of BC cells**. A, qRT-PCR detection of SNHG15 in BC cells transfected with sh-SNHG15-1, sh-SNHG15-2 or sh-NC vector. B, A CCK-8 assay was performed to determine the proliferation of sh-SNHG15-transfected MDA-MB-231 cells. C, A CCK-8 assay was performed to determine the proliferation of sh-SNHG15-transfected MCF7 cells. D and E, A colony formation assay was performed to detect the proliferation ability of SNHG15. F, Flow cytometric analysis of early apoptosis rate in sh-SNHG15 transfected BC cells compared with NC group. *P < 0.05, **P < 0.01.

**Figure 3:**
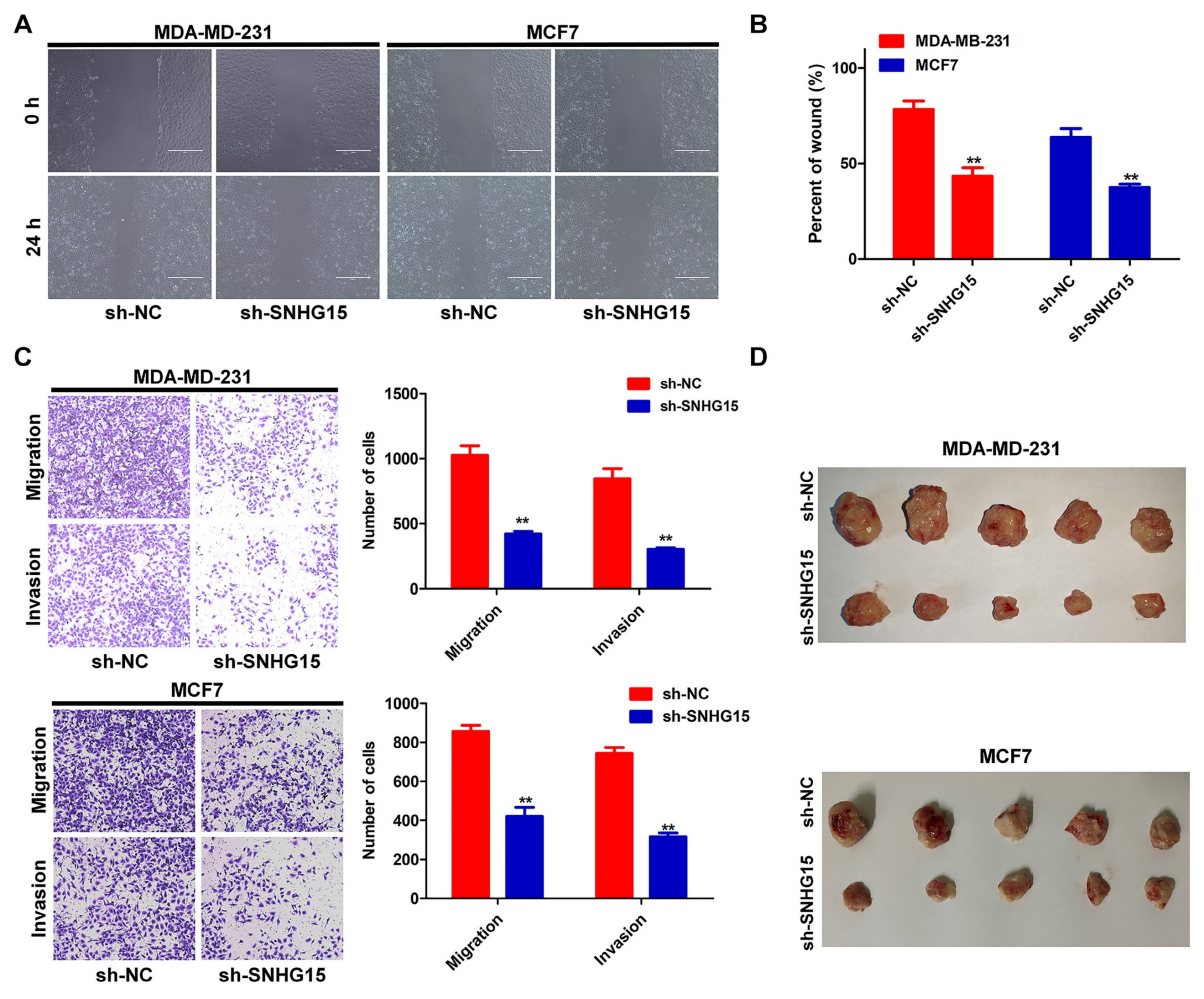
** SNHG15 mediated cell proliferation, migration and invasion of BC cells in vitro and vivo.** A and B, A wound healing assay were performed to investigate changes in cell migration. C, The transwell assay was conducted to detect the migration and invasion of BC cells transfected with sh-NC or sh-SNHG15 vectors. D, Photographs of tumors were provided at 28 days after inoculation. **P < 0.01.

To further investigate the functional role of SNHG15 in BC, sh-SNHG15-1, sh-SNHG15-2 or sh-NC vector was transfected into BC cells. The sh-SNHG15-1 vector was the most efficient shRNA against SNHG15 **(Figure 2A)**. CCK-8 and colony formation experiments showed that the knockdown of SNHG15 inhibited the proliferation of BC cells **(Figure 2B-E)**. FACS apoptosis assay indicated that inhibition of SNHG15 increased early apoptosis in sh-SNHG15 transfected BC cells compared with the NC group **(Figure 2F)**. Likewise, wound healing assay and transwell assays indicated that suppression of SNHG15 could significantly inhibit the migration and invasion of BC cells in comparison with those in the control group **(Figure 3A-C)**. BC cells stably transfected with sh-NC or sh-SNHG15 vector were subcutaneously transplanted into nude mice. Tumor volumes in the sh-SNHG15 group were significantly lower than those in the sh-control group **(Figure 3D)**. In summary, these results indicate that the knockdown of SNHG15 suppresses BC cell proliferation, apoptosis, migration and invasion.

### miR-153-3p is a target of SNHG15 in BC cells

**Figure 4:**
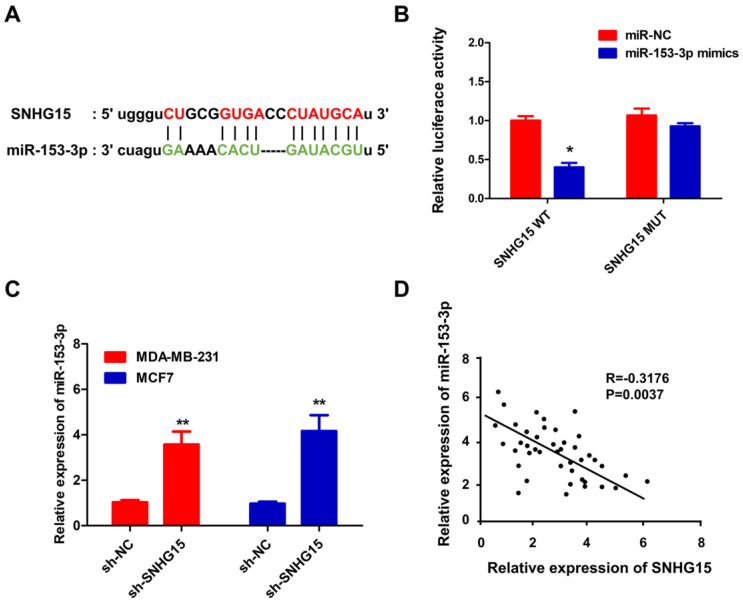
** SNHG15 served as a molecular sponge for miR-153-3p.** A, Schematic diagram showed the putative miR-153-3p binding sites with the SNHG15. B, Luciferase reporter gene assays were performed to measure the luciferase activity. C, qRT-PCR were performed to investigate the changes of miR-153-3p in BC cells transfected with sh-SNHG15 or sh-NC. D, The miR-153-3p and SNHG15 was negatively correlated in BC tissues as determined by linear regression analysis. *P < 0.05, **P < 0.01.

**Figure 5:**
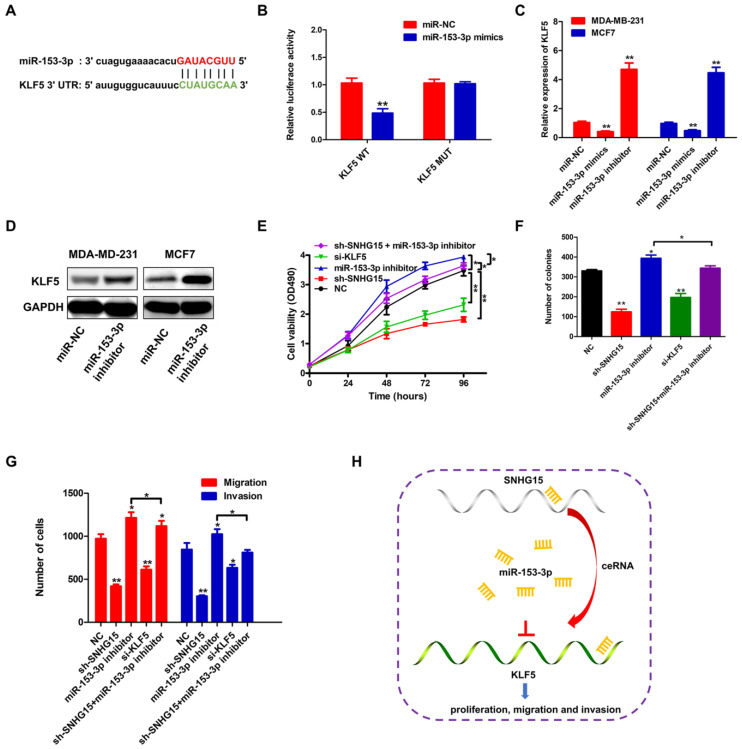
** SNHG15 boosted the progression of BC cells through miR-153-3p/KLF5 signal axis.** A, Schematic diagram showed the putative KLF5 binding sites with the miR-153-3p. B, Luciferase reporter gene assays were performed to measure the luciferase activity. C and D, The relative expression of SNHG15 in BC cells at 36 h after transfection with miR-NC, miR-153-3p mimics or miR-153-3p inhibitor. E and F, A cell proliferation assay and colony formation assay was performed to detect the proliferation ability of the BC cells transfected with NC, sh-SNHG15, miR-153-3p inhibitor, si-KLF5 or sh-SNHG15 + miR-153-3p inhibitor. G, Transwell and matrigel transwell assays were conducted to detect the migration and invasion of BC cells transfected with NC, sh-SNHG15, miR-153-3p inhibitor, si-KLF5 or sh-SNHG15 + miR-153-3p inhibitor. H, The schematic diagram of the mechanism of SNHG15/miR-153-3p/KLF5 signal axis in BC. *P < 0.05, **P < 0.01.

To investigate the possible mechanism of LncRNA SNHG15 in BC biological function, we predicted miRNA binding to lncRNA SNHG15 in several bioinformatics databases (http://mirdb.org/, http://starbase.sysu.edu.cn/, http://www.cuilab.cn/lnctar, http://lncrna.smu.edu.cn/, http://www.targetscan.org), and cross-locked miR-153-3p as its potential target gene. The binding sites between SNHG15 and miR-153-3p were predicted by the bioinformatics database **(Figure 4A)**. Dual-luciferase reporter assays further illustrated the interaction between SNHG15 and miR-153-3p** (Figure 4B)**. To this end, we think miR-153-3p is a target of SNHG15 in BC cells. To further prove this hypothesis, qRT-PCR detections of miR-153-3p were performed in BC cells transfected with sh-SNHG15 or sh-NC and indicated that knockdown of SNHG15 could significantly upregulate miR-153-3p expression **(Figure 4C)**. SNHG15 cDNA was cloned into the luciferase gene and co-transfected with miR-153-3p or miR-NC. Next, a statistically significant negative correlation was verified between miR-153-3p and SNHG15 by linear regression analysis **(Figure 4D)**. Besides, Inhibited expression of miR-153-3p inhibited the proliferative and invasive ability of BC cells, while the abilities were recovered when cells were co-transfected with sh-SNHG15** (Figure 5E-G)**. The results revealed that SNHG15 served as a molecular sponge for miR-153-3p.

### SNHG15 mediated BC progression through miR-153-3p/KLF5 signal axis

To delve deeper into the molecular mechanism underlying miR-153-3p's role in BC, we employed bioinformatic tools to predict its downstream target genes. Our analysis revealed KLF5, a gene implicated in the progression of various tumors, as a potential target with a binding site on miR-153-3p **(Figure 5A)**. Subsequently, the luciferase assay confirmed that the 3′-UTR of wild-type (WT) KLF5 could significantly lower the luciferase activity in the miR-153-3p group without significant influence on the luciferase activity in the miR-NC group **(Figure 5B)**. Additionally, we observed an elevation in both KLF5 mRNA and protein levels in BC cells transfected with a miR-153-3p inhibitor** (Figure 5C, D)**. Cell proliferation assay and colony formation assay indicated the proliferation ability of the sh-SNHG15-transfected BC cells were partially reversed by transfection with miR-153-3p inhibitor **(Figure 5E, F)**. Transwell and matrigel transwell assay indicated that suppression of KLF5 significantly inhibited the migration and invasion of BC cells** (Figure 5G)**. The migration and invasion ability of sh-SNHG15-transfected BC cells were partially reversed by transfection with miR-153-3p inhibitor **(Figure 5G)**. These results indicated that by sponging miR-153-3p, SNHG15 can regulate the expression of KLF5, thus promoting carcinogenesis in BC** (Figure 5H)**.

## Discussion

BC is a malignant tumor with higher morbidity and mortality. At present, the molecular mechanism of BC has not been fully understood. The occurrence and progression of BC was a multistep process involving multiple oncogenes and tumor suppressors. The study of BC related genes is of great significance for finding biomarkers of early BC, formulating targeted treatment strategies and improving patient survival rates. In the past, people always thought that lncRNA is a meaningless transcription by-product. However, with the deepening of research, researchers found that lncRNA plays an important role in the physiological and pathological activities of cells and participates in the occurrence and development of many diseases including tumors 16, 17. In recent years, with the development of high-throughput sequencing technology, increasing lncRNA have been discovered, and many lncRNA are abnormally expressed in BC, which can be used as tumor suppressor genes or oncogenes to participate in the occurrence and development of BC 18-20. At present, some BC-related lncRNA, such as NRAT1, MALAT1, HOTAIR, BC069792, H19, BCAR4 have been found and reported 10, 12, 14, 21-25. Nevertheless, the research on the regulation mechanism of lncRNA in BC is still in its infancy, and the role of lncRNA in BC is not yet fully understood.

Human lncRNA SNHG15 is located at chromosome 7p13 with a length of 860 bp. It has been reported in the literature that SNHG15 is highly expressed in lung cancer, gastric cancer, liver cancer, colon cancer, cervical cancer and ovarian cancer, which is related to the prognosis of patients, and plays the role of oncogenes in the occurrence and development of malignant tumors 26-30. The highly expressed SNHG15 binds to the C-terminal zinc finger domain of Slug, which promotes the growth and metastasis of colon cancer 31. It is reported that SNHG15 promotes the progression of renal cell carcinoma by regulating the NF-κB signaling pathway. However, there are few reports about SNHG15 in BC 32. In this study, we found that SNHG15 was highly expressed in human BC, and upregulated SNHG15 was correlated with large tumor size and advanced TNM stage. Meanwhile, it was found that the expression of SNHG15 in BC cell lines was higher than that in normal breast cells. Knockdown of SNHG15 suppresses BC cell proliferation, apoptosis, migration and invasion. The results indicate that SNHG15 may also play a role in promoting the occurrence and development of BC.

LncRNA can regulate gene expression at different levels, and its specific mechanisms can be summarized as epigenetic regulation, transcription regulation, post-transcriptional regulation and lncRNA-miRNA-mRNA regulation 33, 34. To further understand the possible mechanism of SNHG15 in BC biological function, we predicted miRNA-153-3p binding to SNHG15 using bioinformatics databases. Subsequently, the association of cell function assay and double luciferase reporter proved that miR-153-3p was the target of SNHG15 in BC cells. SNHG15 regulates BC cell functions by acting as a competitive endogenous RNA for miRNA-153-3p. Similarly, recent research shows that SNHG15 acted as a miR-153-3p sponge in hypoxic-ischemic encephalopathy 35. This also further corroborates our experimental results, that is, miR-153-3p was the target of SNHG15.

MiRNA exerts its biological influence through interactions with downstream target gene mRNA, as documented in previous studies 36, 37. To further understand the underlying molecular mechanism of SNHG15-miR-153-3p in BC, we predicted the downstream target genes of miR-153-3p using bioinformatic tools. KLF5 is a member of the KLF family, also known as IKLF (intensive-enriched Krupp El-like factor) and BTEB2 (basic transcription element-binding protein 2) 38. Recent research shows that KLF5 has a potential oncogene function in BC 39, 40. We observed that miR-153-3p targeted and negatively regulated KLF5 function in BC. This is consistent with the experimental results in some other disease models 41, 42. Additional experiments further supported that SNHG15's oncogenic activity in BC is partly mediated through the miR-153-3p/KLF5 axis.

## Conclusions

In summary, this study has revealed an association between altered lncRNA SNHG15 levels and the progression of BC. Specifically, SNHG15 appears to drive proliferation and metastasis by acting as a sponge for miR-153-3p, thereby modulating KLF5 expression. These findings suggest that SNHG15 could serve as a promising biomarker and therapeutic target in the management of BC.

## Figures and Tables

**Table 1:** Correlation of the expression of SNHG15 with clinicopathologic features

Clinicopathologic features	n (%)	Relative expression of SNHG15	P-value
Age (years)			0.643
< 60	18 (42.86)	6.34 ± 3.23	
≥ 60	24 (57.14)	8.21 ± 4.47	
Gender			-
Male	0 (0)	-	
Female	42 (100)	7.40 ± 4.06	
Tumor size			0.002 **
≤ 20 mm	15 (35.71)	4.78 ± 3.06	
> 20 mm	27 (64.29)	8.96 ± 5.03	
TNM stage			0.007 **
I, II	26 (61.90)	4.64 ± 2.78	
Ⅲ, Ⅳ	16 (38.10)	9.23 ± 5.67	

Data are presented as the mean ± SEM. *P<0.05, **P<0.01.

## References

[1] Siegel RL, Kratzer TB, Giaquinto AN, Sung H, Jemal A (2025). Cancer statistics, 2025. CA Cancer J Clin.

[2] Giaquinto AN, Sung H, Newman LA, Freedman RA, Smith RA, Star J (2024). Breast cancer statistics 2024. CA Cancer J Clin.

[3] Qi J, Li M, Wang L, Hu Y, Liu W, Long Z (2023). National and subnational trends in cancer burden in China, 2005-20: an analysis of national mortality surveillance data. Lancet Public Health.

[4] Loibl S, Poortmans P, Morrow M, Denkert C, Curigliano G (2021). Breast cancer. Lancet (London, England).

[5] Yin X, Wang P, Yang T, Li G, Teng X, Huang W (2020). Identification of key modules and genes associated with breast cancer prognosis using WGCNA and ceRNA network analysis. Aging.

[6] Henikoff S, Zheng Y, Paranal RM, Xu Y, Greene JE, Henikoff JG (2025). RNA polymerase II at histone genes predicts outcome in human cancer. Science.

[7] Kung JT, Colognori D, Lee JT (2013). Long noncoding RNAs: past, present, and future. Genetics.

[8] Yao RW, Wang Y, Chen LL (2019). Cellular functions of long noncoding RNAs. Nature cell biology.

[9] Schmitt AM, Chang HY (2016). Long Noncoding RNAs in Cancer Pathways. Cancer cell.

[10] Park MK, Zhang L, Min KW, Cho JH, Yeh CC, Moon H (2021). NEAT1 is essential for metabolic changes that promote breast cancer growth and metastasis. Cell Metab.

[11] Chen F, Chen J, Yang L, Liu J, Zhang X, Zhang Y (2019). Extracellular vesicle-packaged HIF-1α-stabilizing lncRNA from tumour-associated macrophages regulates aerobic glycolysis of breast cancer cells. Nature cell biology.

[12] Kim J, Piao HL, Kim BJ, Yao F, Han Z, Wang Y (2018). Long noncoding RNA MALAT1 suppresses breast cancer metastasis. Nature genetics.

[13] Liu Y, Zhang P, Wu Q, Fang H, Wang Y, Xiao Y (2021). Long non-coding RNA NR2F1-AS1 induces breast cancer lung metastatic dormancy by regulating NR2F1 and ΔNp63. Nature communications.

[14] Crudele F, Bianchi N, Reali E, Galasso M, Agnoletto C, Volinia S (2020). The network of non-coding RNAs and their molecular targets in breast cancer. Molecular cancer.

[15] Jin H, Du W, Huang W, Yan J, Tang Q, Chen Y (2021). lncRNA and breast cancer: Progress from identifying mechanisms to challenges and opportunities of clinical treatment. Molecular therapy Nucleic acids.

[16] Marchese FP, Raimondi I, Huarte M (2017). The multidimensional mechanisms of long noncoding RNA function. Genome biology.

[17] Zhang Y, Wei S, Chen Z, Xu R, Li SR, You L (2024). LncRNA FAISL Inhibits Calpain 2-Mediated Proteolysis of FAK to Promote Progression and Metastasis of Triple Negative Breast Cancer. Adv Sci (Weinh).

[18] Sideris N, Dama P, Bayraktar S, Stiff T, Castellano L (2022). LncRNAs in breast cancer: a link to future approaches. Cancer gene therapy.

[19] Han X, Mo J, Yang Y, Wang Y, Lu H (2022). Crucial Roles of LncRNAs-Mediated Autophagy in Breast Cancer. International journal of medical sciences.

[20] Raju GSR, Pavitra E, Bandaru SS, Varaprasad GL, Nagaraju GP, Malla RR (2023). HOTAIR: a potential metastatic, drug-resistant and prognostic regulator of breast cancer. Mol Cancer.

[21] Ren Y, Jia HH, Xu YQ, Zhou X, Zhao XH, Wang YF (2018). Paracrine and epigenetic control of CAF-induced metastasis: the role of HOTAIR stimulated by TGF-ß1 secretion. Molecular cancer.

[22] Wang J, Xie S, Yang J, Xiong H, Jia Y, Zhou Y (2019). The long noncoding RNA H19 promotes tamoxifen resistance in breast cancer via autophagy. Journal of hematology & oncology.

[23] Peperstraete E, Lecerf C, Collette J, Vennin C, Raby L, Völkel P (2020). Enhancement of Breast Cancer Cell Aggressiveness by lncRNA H19 and its Mir-675 Derivative: Insight into Shared and Different Actions. Cancers.

[24] Peng R, Cao J, Guo Q, Sun Q, Xu L, Xie X (2021). Variant in BCAR4 gene correlated with the breast cancer susceptibility and mRNA expression of lncRNA BCAR4 in Chinese Han population. Breast cancer (Tokyo, Japan).

[25] Zhang Y, Dong X, Guo X, Li C, Fan Y, Liu P (2023). LncRNA-BC069792 suppresses tumor progression by targeting KCNQ4 in breast cancer. Mol Cancer.

[26] Saeinasab M, Bahrami AR, González J, Marchese FP, Martinez D, Mowla SJ (2019). SNHG15 is a bifunctional MYC-regulated noncoding locus encoding a lncRNA that promotes cell proliferation, invasion and drug resistance in colorectal cancer by interacting with AIF. Journal of experimental & clinical cancer research: CR.

[27] Yang J, Yang M, Lv H, Zhou M, Mao X, Qin X (2022). lncRNA SNHG15 Induced by SOX12 Promotes the Tumorigenic Properties and Chemoresistance in Cervical Cancer via the miR-4735-3p/HIF1a Pathway. Oxidative medicine and cellular longevity.

[28] Fang XY, Pan HF, Leng RX, Ye DQ (2015). Long noncoding RNAs: novel insights into gastric cancer. Cancer letters.

[29] Shuai Y, Ma Z, Lu J, Feng J (2020). LncRNA SNHG15: A new budding star in human cancers. Cell proliferation.

[30] Damaskos C, Garmpis N, Dimitroulis D, Garmpi A, Diamantis E, Sarantis P (2022). The Role of SNHG15 in the Pathogenesis of Hepatocellular Carcinoma. Journal of personalized medicine.

[31] Chen S, Shen X (2020). Long noncoding RNAs: functions and mechanisms in colon cancer. Mol Cancer.

[32] Ahmad S, Abbas M, Ullah MF, Aziz MH, Beylerli O, Alam MA (2022). Long non-coding RNAs regulated NF-κB signaling in cancer metastasis: Micromanaging by not so small non-coding RNAs. Semin Cancer Biol.

[33] Zhang Y, Tao Y, Liao Q (2018). Long noncoding RNA: a crosslink in biological regulatory network. Briefings in bioinformatics.

[34] Paraskevopoulou MD, Hatzigeorgiou AG (2016). Analyzing MiRNA-LncRNA Interactions. Methods in molecular biology (Clifton, NJ).

[35] Fu J, Huang Y, Xian L (2022). LncRNA SNHG15 regulates hypoxic-ischemic brain injury via miR-153-3p/SETD7 axis. Histology and histopathology.

[36] Pouya FD, Rasmi Y, Gazouli M, Zografos E, Nemati M (2022). MicroRNAs as therapeutic targets in breast cancer metastasis. Drug delivery and translational research.

[37] Chan JJ, Tay Y (2018). Noncoding RNA:RNA Regulatory Networks in Cancer. International journal of molecular sciences.

[38] McConnell BB, Yang VW (2010). Mammalian Krüppel-like factors in health and diseases. Physiological reviews.

[39] Jia L, Zhou Z, Liang H, Wu J, Shi P, Li F (2016). KLF5 promotes breast cancer proliferation, migration and invasion in part by upregulating the transcription of TNFAIP2. Oncogene.

[40] Jiang D, Qiu T, Peng J, Li S, Tala, Ren W (2022). YB-1 is a positive regulator of KLF5 transcription factor in basal-like breast cancer. Cell death and differentiation.

[41] Wang KF, Shi ZW, Dong DM (2021). CircATRNL1 protects against osteoarthritis by targeting miR-153-3p and KLF5. International immunopharmacology.

[42] Liu R, Chen H, Zhao P, Chen CH, Liang H, Yang C (2020). Mifepristone Derivative FZU-00,003 Suppresses Triple-negative Breast Cancer Cell Growth partially via miR-153-KLF5 axis. International journal of biological sciences.

